# Attenuated and Protease-Profile Modified Sendai Virus Vectors as a New Tool for Virotherapy of Solid Tumors

**DOI:** 10.1371/journal.pone.0090508

**Published:** 2014-03-05

**Authors:** Martina Zimmermann, Sorin Armeanu-Ebinger, Sascha Bossow, Johanna Lampe, Irina Smirnow, Andrea Schenk, Sebastian Lange, Thomas S. Weiss, Wolfgang Neubert, Ulrich M. Lauer, Michael Bitzer

**Affiliations:** 1 Department of Internal Medicine I, Medical University Hospital, Tübingen, Germany; 2 University Childrens Hospital, Tübingen, Germany; 3 Department of Translational Oncology, National Center for Tumor Diseases, Heidelberg, Germany; 4 Institute for Molecular Medicine Finland FIMM, Helsinki, Finland; 5 Medizinische Klinik, Klinikum rechts der Isar, Technische Universität, München, Germany; 6 Center for Liver Cell Research, Department of Pediatrics and Adolescent Medicine, University of Regensburg Hospital, Regensburg, Germany; 7 Max-Planck-Institute for Biochemistry, Department Molecular Virology, Martinsried Germany; University of Rochester Medical Center, United States of America

## Abstract

Multiple types of oncolytic viruses are currently under investigation in clinical trials. To optimize therapeutic outcomes it is believed that the plethora of different tumor types will require a diversity of different virus types. Sendai virus (SeV), a murine parainfluenza virus, displays a broad host range, enters cells within minutes and already has been applied safely as a gene transfer vector in gene therapy patients. However, SeV spreading naturally is abrogated in human cells due to a lack of virus activating proteases. To enable oncolytic applications of SeV we here engineered a set of novel recombinant vectors by a two-step approach: (i) introduction of an ubiquitously recognized cleavage-motive into SeV fusion protein now enabling continuous spreading in human tissues, and (ii) profound attenuation of these rSeV by the knockout of viral immune modulating accessory proteins. When employing human hepatoma cell lines, newly generated SeV variants now reached high titers and induced a profound tumor cell lysis. In contrast, virus release from untransformed human fibroblasts or primary human hepatocytes was found to be reduced by about three log steps in a time course experiment which enables the cumulation of kinetic differences of the distinct phases of viral replication such as primary target cell infection, target cell replication, and progeny virus particle release. In a hepatoma xenograft animal model we found a tumor-specific spreading of our novel recombinant SeV vectors without evidence of biodistribution into non-malignant tissues. In conclusion, we successfully developed novel tumor-selective oncolytic rSeV vectors, constituting a new tool for virotherapy of solid tumors being ready for further preclinical and clinical development to address distinct tumor types.

## Introduction

One of the most important barriers that limit the successful treatment of cancer today is constituted by the presence of primary or by the development of secondary resistance phenomena. Thus, the tools to hit cancer cells should contain as much as possible fundamentally different target options. An evolving new field in clinical oncology is the application of conditionally replicating viruses that selectively destroy tumor cells, so called oncolytic viruses [1], [2], [3], [4]. Several different mechanisms leading to a virus-induced, cancer cell-specific killing have been found, such as activation of viral particles by cancer-specific proteases, entry through cancer cell-specific receptors or exploiting specific defects of cancer cells [5]. An example for such tumor cell defect is the inability of most tumor cells to produce or to respond to interferon (IFN) after viral infection [6], [7], [8]. As a consequence, IFN-sensitive viruses preferentially replicate in cancer cells while normal cell types and tissues are able to launch a powerful counterattack [9]. Virotherapy based clinical studies are currently undertaken for several different DNA and RNA viruses [3], [10]. Due to the diversity of the so far established viral systems and the plethora of different tumor types that have to be addressed, it is challenging to identify or develop distinct oncolytic viruses that are most suitable for a subset of tumor entities.

In this context, some of the attractive features of Paramyxoviruses with negative stranded RNA genomes are an exclusively cytoplasmic replication without any risk for DNA integration, a strong and adjustable gene expression of virally encoded genes, the determination of host cell tropism by viral surface glycoproteins, and well established genetic manipulation procedures [5], [11], [12], [13]. The feasibility of an application of the paramyxoviruses measles vaccine virus (MeV) and Newcastle disease virus (NDV) to cancer patients has already been shown as a proof-of-principle in early clinical trials [14], [15].

Unmodified wild type Sendai virus (murine parainfluenzavirus type I, SeV), another well-known member of this virus family, was even used as a vaccine vector against hPIV1 and has been demonstrated to be safe in clinical trials [16]. Additionally, a first-in-man application of recombinant Sendai virus has been reported very recently as a vector for gene delivery of human FGF-2 to treat peripheral arterial disease and was shown to be safe and well tolerated [17].

Basically, SeV as a prototype paramyxovirus has been intensively investigated and characterized on a molecular level. SeV displays a very broad host range because it can use ubiquitously expressed sialic acid containing ganglioside receptors for cell entry via SeV HN protein interaction [18], [19], [20], [21], leading to a rapid uptake into the cytoplasm within minutes [11]. These characteristics are of specific interest for broad range antitumoral approaches, because cell entry occurs independently of the expression of specialized receptors on the tumor cell surface. However, cleavage of the viral precursor fusion protein F_0_ into the active subunits F_1_ and F_2,_ which is essential for virus entry into the target cell, takes place by proteases, which by nature are only present in the murine respiratory tract (trypsin-like proteases [22]). This fact severely limits the replication of SeV to only a single round of infection in all other tissues, which *per se* prevents any substantial oncolytic capacity.

Driven by the attractive profile of SeV, efforts have been undertaken to purposefully engineer wild-type SeV for application in oncology. In contrast to our approach here, those efforts tried to restrict the cleavability of the viral fusion protein F rigorously to predefined tumor secreted proteases, such as matrix-metalloproteinases, combined with an enhancement of the SeV-inherent syncytia formation capacity by deletion of the structural matrix gene M or truncation of the fusion protein F [23], [24], [25]. However, that strategy restricts the usage of SeV as a potential antitumor agent strictly to the injection site and to tumors that overexpress individual proteases, which might favor the unwanted selection of tumor cells with an altered protease composition.

Therefore, we now developed a completely new SeV based oncolytic system in a two-step approach. First, we introduced a new cleavage-site into the viral fusion protein that can be cleaved by ubiquitously available proteases to expand viral replication to tumor cells. Second, we attenuated SeV particles to a low replication capacity in non-malignant cell types by deleting accessory viral proteins that are well known to harbor e. g. interferon-antagonizing properties [26], [27], [28], [29], [30]. Thus, our newly generated SeV particles are (i) able to benefit from the broad and attractive host range and rapid cellular uptake of SeV, (ii) have the capacity to generate complete infectious viral particles *in vivo*, and (iii) harbor a strong attenuation by repressing any relevant viral replication in untransformed or non-malignant cell types.

## Materials and Methods

### Ethics statements

Primary human hepatocytes (PHH) from different donors were provided via the charitable state controlled foundation Human Tissue & Cell Research HTCR (http://www.htcr.de) with written informed patient's consent approved by the local ethical committee of the University of Regensburg, Germany.

All animal experiments were performed in agreement with the German animal welfare act. The protocol (M 8/09) was approved by the local ethics committee for animal experimentation (Regierungspräsidium Tübingen, Baden-Württemberg, Germany).

### Generation of recombinant SeV

For construction of a full-length SeV cDNA with mutations in SeV P-gene, plasmid pSVV10 coding for the SeV genome of strain Fushimi and also harboring an enhanced green fluorescent protein (EGFP) at leader position was used (manufactured by Guy Ungerechts, UKT Tübingen). Different plasmid variants with P-gene mutations were generated via mutagenesis. To perform mutagenesis (QuikChange Multi Site-Directed Mutagenesis Kit, Stratagene, Amsterdam, the Netherlands), the cloning relevant sequence of the SeV vector pSVV10 (Guy Ungerechts, UKT Tübingen) was subcloned using *Sph*I and *Eco*RI restriction enzymes into the cloning vector pSL1180 (Amersham, Munich, Germany). To insert single point mutations into the SeV P-gene sequence, three different mismatch primers were used: SeVC_ko_5′-CGCATGGATCAAGACGCCTTCATTCTAAAAGAAGATTCTGAAGTAGAGAGG-3′, SeVY_ko_ 5′-CTCTCGGACGTTATCGGATTCCTCGACGCTGTCCTG-3′, SeVV_ko_ 5′-GACTCAACAAAGAAAGGCATAGGTGAGAACACATCATCTATG-3′. Resulting mutated sequences were inserted via *Sph*I and *Eco*RI into a pSVV13ΔFΔHN SeV cDNA genome coding plasmid (strain Fushimi, Guy Ungerechts, UKT Tübingen), equivalent to vector pSVV10 but without F and HN genes. To reconstitute the full-length SeV cDNA, the lacking sequences (*Eco*RI-*Eco*RI fragment) with fragments of the P- and L-gene, the M- and the F-gene with F-protein cleavage-site from Newcastle disease virus (Fmut; RRQKR instead of VPQSR) of the SeV plasmid pRSldEGFP Fmut (strain Fushimi; Sabine Schlecht, Munich, Germany) were inserted. All P-gene mutations were double checked via sequencing. More detailed information is provided upon request. Recombinant Sendai viruses (rSeV) were rescued and propagated as described before [31]. Propagation of control virus SeV D52 (also of strain Fushimi), representing SeV-P/SeV-F wild-type genes, was performed in serum free medium (DMEM) with 3 µg/ml acetyl trypsin. Viral titers were determined via 50% tissue culture infective dose titration (TCID_50_) on Vero cells [32].

### Cells

African green money kidney cells (Vero, DSMZ, Braunschweig, Germany), human lung fibroblasts (MRC-5, ECACC, Porton Village, UK), and human hepatoma cell lines (Hep3B, ECACC; PLC/PRF/5, ECACC; HuH7, Riken Gene Bank, Tsukuba, Japan) were cultured in Dulbecco's modified Eagle's medium (DMEM with 2 mmol/l L-glutamine; Biochrom AG, Berlin, Germany) containing 10% fetal calf serum (FCS, PAA Laboratories GmbH, Pasching, Austria). All cells were tested to be negative for mycoplasma contamination. Primary human hepatocytes (PHH; provided by Dr. Thomas Weiss, UKR) from different donors were isolated and cultured as described before [33] according to the guidelines and informed consent of the charitable state-controlled Human Tissue & Cell Research Foundation HTCR (http://www.htcr.de).

### Virus Growth curves

Cells were seeded in a 12-well plate (cell lines: 1×10^5^ cells per well; PHH: 1.2×10^5^ cells per cm^2^) and infected with 5×10^3^ Sendai virus particles (TCID_50_) in Opti-MEM (Invitrogen, Darmstadt, Deutschland). After an incubation time of 3 h, the inoculum was removed and the cells were washed with PBS twice. One ml fresh DMEM medium supplemented with 5% FCS was added and supernatants were harvested immediately (3 hpi) or after 24, 48, 72 and 96 hpi. The remaining cells were washed with PBS twice and scraped in 1 ml DMEM containing 5% FCS. Supernatants and lysates were frozen and stored at -80°C. After controlled thawing for exactly 3 min at °C, vortexing and centrifugation, amounts of infectious virus particles in supernatants and cell lysates were determined via TCID_50_ titration.

### Determination of cytotoxicity

To determine virus mediated cytotoxicity, the amount of remaining cell mass and the loss of cellular integrity were determined via sulforhodamine B (SRB [34]) and lactate dehydrogenase (LDH; P-mono, Analyticon Biotechnologies AG, Lichtenfels, Germany) assays. For time course viability analysis, the CytoTox-Glo Assay™ (Promega, Mannheim, Germany) was used. The cells were seeded in 96-well plates and infections were performed at MOIs of 0.1 and 1. Control cells were chemically treated with 0.1% TX (Triton X-100) to induce maximum grade destruction. At each time point post infection (0 h, 24 hpi, 48 hpi, 72 hpi, 96 hpi) supernatants were removed, cells were washed with PBS, followed by addition of 50 µl of lysis buffer. After 15 min of incubation, the resulting luminescence was quantified.

For SRB and LDH assays, cells were seeded in 24-well plates (cell lines: 5×10^4^ cells per well; PHH: 3×10^5^ cells per well) and were infected with a multiplicity of infection (MOI) 0.01 and 0.1 in Opti-MEM in triplicates, each. Mock infected control cells were used as controls. Pictures (fluorescence or phase contrast) were taken every 24 h to track the infections (microscope: Olympus IX50, Hamburg, Germany; software: Analysis 3.1, Soft Imaging System GmbH, Münster, Germany). To quantify the remaining cell mass, cells were fixed with cold trichloroacetic acid (TCA, 10% w/v) and stained with SRB-staining solution (0.4% w/v sulforhodamine B in 1% acetic acid). The protein bound SRB dye was resolved in Tris (10 mM, pH 10.5) and the optical density was measured at 550 nm. Remaining cell mass was calculated by normalization of optical density between uninfected and virus treated samples.

For LDH assay, 72 h post infection the amount of lactate dehydrogenase (LDH) was measured in both supernatants and the remaining cell masses. For this purpose, reaction buffer (pyruvate and NADH, P-mono Kit, Analyticon Biotechnologies AG, Lichtenfeld, Germany) was added to culture supernatant and lysates of remaining cells (lysis buffer: PBS/0.1% Triton-X). The decrease of NADH was measured at 340 nm. The cellular lysis was calculated by the ratio of LDH activity in supernatant to total LDH activity per each well corrected by LDH activity in the fresh culture media. The virus-induced lysis was related to cellular lysis in mock infected cultures.

### *In vivo* studies

All animal experiments were performed in agreement with the German animal welfare act. The protocol was approved by the local ethics committee for animal experimentation. PLC/PRF/5 cells (1×10^7^) were injected into the right flank of Balb/c-nude mice (CanN.Cg-Foxn1nu/Crl, Charles River, Sulzfeld, Germany). Every two to three days animal weights and tumor volumes were measured. After the tumor reached a size of more than 200 mm^3^, 1×10^7^ TCID_50_ SeV virus particles were injected intratumorally (2–3 animals per group). Two days after virus injection, animals were sacrificed and liver, spleen, heart, lung and tumor were harvested using fresh scissors for each organ. Parts of the organs were snap frozen in 1 ml Opti-MEM or transferred into formalin (4%, Fischar GmbH & Co. KG, Saarbrücken, Germany) or RNA later (Ambion, Life Technologies GmbH, Darmstadt, Germany) for further analysis.

### Histology

Formalin fixed tissue samples were dehydrated and embedded in paraffin. Microtome sections (4 µm) were prepared and the virally expressed GFP was detected applying a specific antibody (anti GFP, ab290, Abcam, Cambridge, UK) and the Vectastain rabbit ABC Kit (Vector Laboratories, Burlingame, CA, USA). Haematoxylin (Gill; Roth, Karlsruhe, Germany) was used for counterstaining.

### Isolation of SeV virus particles from primary tissues

Frozen tissues (in Opti-MEM, a quarter of each organ) were thawed and pressed through a cell strainer (40 µm) using a sterile plunger. The cellular debris was sedimented by centrifugation (12,000×g) and the supernatant was transferred to Vero cells in 6-well plates to identify even minimal amounts of infectious viral particles. 1 day post infection, medium was changed to DMEM/2 mmol/l L-glutamine with 5% FCS for rSeV Fmut and DMEM/2 mmol/l L-glutamine with 3 µg/ml acetyl trypsin for SeV D52. For virus quantification, frozen tissue sections were thawed at 37°C in 1 ml OptiMEM and pressed though a cell strainer. After centrifugation, the amount of infectious particles in the supernatant was quantified via TCID_50_ Titration.

## Results

### Generation of recombinant Sendai viruses

**Figure 1:**
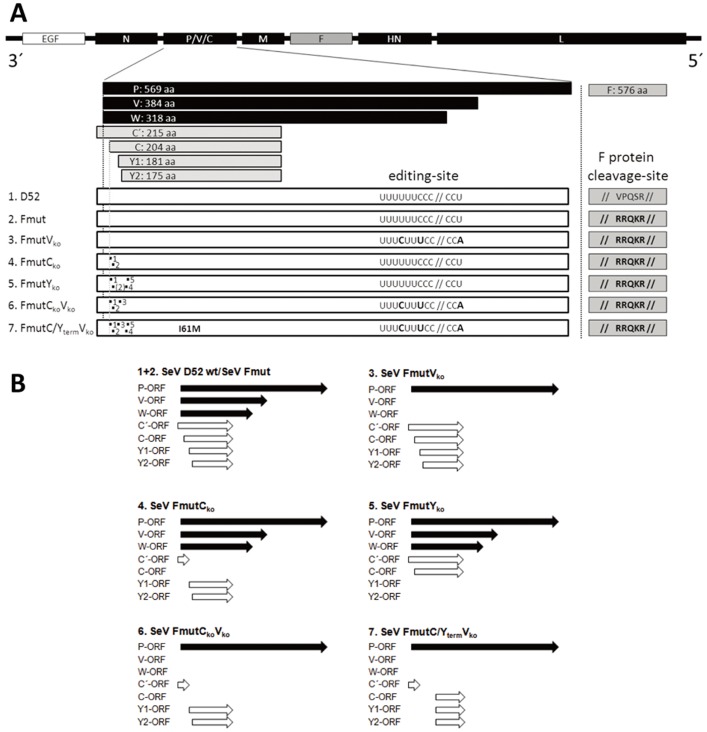
Generation of recombinant Sendai virus variants. **(A)** Schematic representation of SeV genomes and the protein sequence of the SeV-F protein cleavage-site of newly generated SeV virus variants. At the 3′-end all variants encode a reporter gene for enhanced green fluorescent protein (EGFP). In contrast to the P and F gene wild-type variant (D52), in all SeV Fmut variants the wild-type F protein cleavage-site (VPQSR) was replaced by the oligobasic cleavage-site of Newcastle disease virus F protein (RRQKR). The seven wild-type P gene encoded proteins are a result of multiple open reading frames (ORF; C′, C, Y1, Y2) and RNA editing, respectively, leading to a frame shift and thus to the V or W ORF's. For attenuation in non-malignant cell types, different mutations were introduced in C (ORFs; C′-, C-, Y1-, Y2) or V/W-ORFs. For the V_ko_ variants (no V and no W proteins), mutations of the editing-site within the P frame were introduced without changing the amino acid sequence of the P protein. Thus, P protein but no truncated P protein variants (V and W proteins) can be synthesized. For the C protein deficient variants (C_ko_: no C′ and C proteins; Y_ko_: no Y1 and Y2 proteins), inserted point mutations are marked by numbers from 1-5 (1: M1T, 2: L5stop, 3: L11stop, 4: M24T, 5: M30T; numbers refer to amino acid position in C/Y-frame whereas 1 refers to the initiator methionine of the C protein). **(B)** Schematic overview of functional ORFs from the P gene of different SeV variants. The SeV P-gene wild-type expression pattern is shown for D52 and Fmut (1.+2.).

For the generation of SeV particles that conditionally replicate in tumor cells and thereby induce a profound oncolysis, a two-step-approach was performed: first, the natural protease cleavage-site of SeV-F protein was replaced by a cleavage-site which can be cleaved by ubiquitously available proteases followed by an artificial attenuation of SeV that selectively exploits tumor cell defects within the innate defense system. Basically, the spread of SeV wild type infection is restricted to the presence of trypsin-like proteases that are able to activate the viral precursor fusion. Therefore, the monobasic F cleavage-site (VPQSR) was replaced by a NDV-derived oligobasic cleavage-site (RRQKR), a substrate for ubiquitously expressed furin-like proteases, generating the virus variant Fmut (Figure 1A).

As a second step we addressed the reaction pattern of SeV host cells to viral infection. It is well known that viral replication induces cellular innate defense mechanisms such as dsRNA detection during RNA virus replication, which triggers the expression and secretion of IFN α/β, followed by an IFN α/β receptor (IFNAR) mediated activation of the JAK/STAT pathway and the expression of IFN-stimulated genes (ISG [35]). To overcome cellular innate defense mechanisms, some viruses express antagonizing proteins [36]. SeV accessory proteins (C′, C, Y1, Y2, V, W) are well known to harbor such an IFN-antagonizing capacity [29], [30], [37] and recombinant viruses without these proteins are strongly attenuated *in vitro* and *in vivo* [28], [38]. It is further assumed that these viral proteins also interfere with other unspecific cellular defense mechanisms. Thus, to exploit tumor-specific defects in viral defense mechanisms, we introduced point mutations in the P gene of the SeV cDNA genome without altering the amino acid sequence of the P protein but selectively disturbing the transcription of C/Y or V/W proteins. Altogether, five SeV P gene variants were newly generated (Figure 1A/B, variants 3–7). Viruses with at least two C or Y proteins (Figure 1, SeV FmutV_ko_, SeV FmutC_ko_ and SeV FmutC_ko_V_ko_) could easily be propagated and displayed a stable genotype, which was confirmed by sequencing the region of interest. In contrast, although the rescue of variants missing all four C proteins (C′, C, Y1, Y2) was successful, it was not possible to produce stable high titer preparations of these viruses as already observed by others [39], [40]. During the initial propagation of our new SeV variants lacking all four C proteins (SeV FmutC_ko_Y_ko_ and SeV FmutC_ko_Y_ko_V_ko_), in which the expression of V/W proteins was omitted too, point mutations occurred which led to two new genotypes (Figure 1):

(i) a reversion of an inserted STOP codon in the C/Y frame of a preliminary SeV FmutC_ko_Y_ko_ variant (Fig 1A, point mutation 2) enabled the translation of either C′- and C-mRNA, resulting in a SeV FmutY_ko_ variant, and (ii) the development of a single point mutation in a previous SeV FmutC_ko_Y_ko_V_ko_ variant, which led to a late C/Y frame start codon (I61M) enabling the expression of a C-terminal C/Y protein (SeV FmutC/Y_term_V_ko_ variant). Since no high performance (commercial) antibodies being specific for these accessory SeV proteins are available, expression analysis via western blotting was found to be rather difficult. In this line, also low amounts of these proteins and an expression pattern which seems to vary throughout the virus replication cycle contributed to these difficulties.

To investigate whether these additionally acquired mutations are stable, they were further propagated and sequenced after three propagation cycles. Notably, the point mutations did not change during this propagation procedure, thereby reflecting a stable viral genotype for SeV FmutY_ko_ and for SeV FmutC/Y_term_V_ko_. Thus, six recombinant SeV Fmut variants (Figure 1) and a variant representing the wild-type P and F gene (SeV D52) were available for testing of their possible oncolytic properties.

### Tumor selective high titer replication and rapid spread of newly generated SeV variants in malignant human hepatoma cell lines

**Figure 2:**
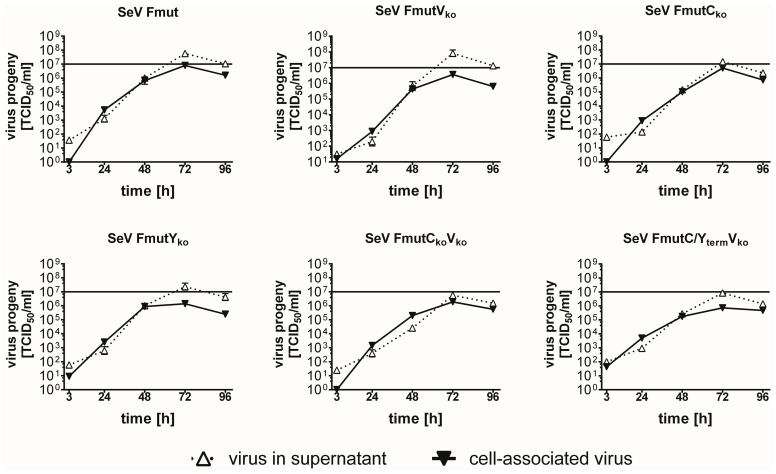
Growth kinetics of newly generated SeV variants in Vero producer cells. Growth kinetics of six different recombinant SeV viruses over a 96×10^7^ TCID_50_ was inserted for better orientation.

**Figure 3:**
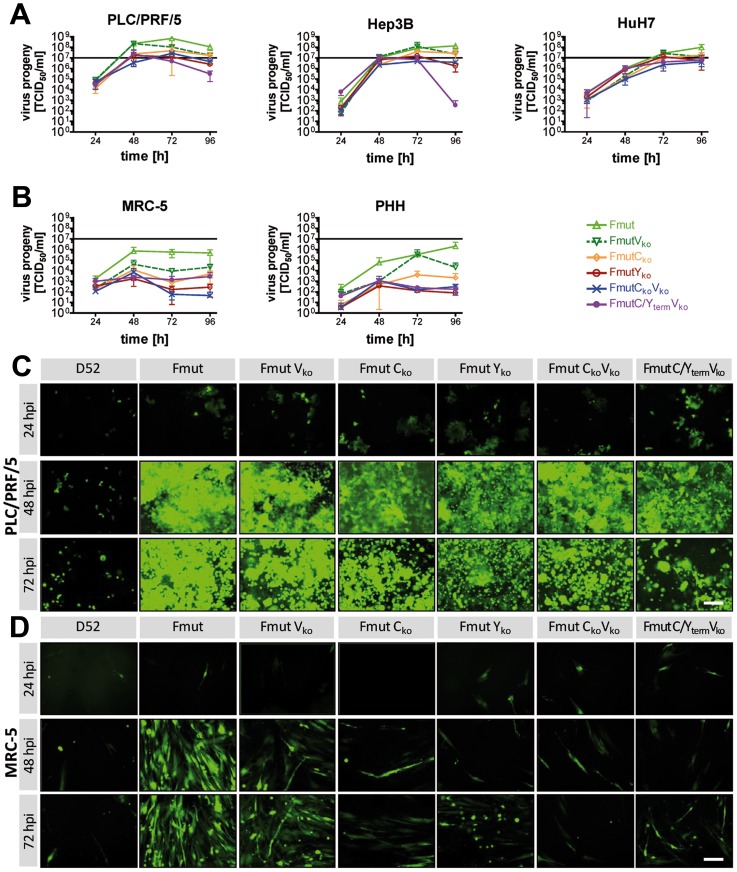
Growth kinetics and spreading of newly generated SeV variants in different cell types. **(A+B)** Growth kinetics of six different recombinant SeV viruses over a 96×10^7^ TCID_50_ is depicted. **(A)** Malignant human hepatoma cells PLC/PRF/5, Hep3B and HuH7. **(B)** Non-malignant MRC-5 fibroblasts and primary human hepatocytes (PHH) from three different donors. **(C+D)** Detection of EGFP reporter protein expression over a 72 h observation period by fluorescence microscopy as a surrogate marker for viral replication and spread to neighboring cells. Size bar: 200 µm. **(C)** Infection of PLC/PRF/5 hepatoma cells. **(D)** Infection of MRC-5 human fibroblast cells.

After successful rescue of the different recombinant viruses, replication in Vero cells demonstrated a trypsin-independent propagation for all SeV Fmut variants (Figure 2). As an *in vitro* tumor model we chose three human hepatoma cell lines, namely PLC/PRF/5, Hep3B and HuH7. All newly generated SeV Fmut variants were able to replicate efficiently in these human tumor cells (Figure 3A). Notably, SeV Fmut and SeV FmutV_ko_ produced even higher amounts of viral progeny particles in the hepatoma cell supernatants compared to Vero cell supernatants (Figure 2) with a maximum amount at 48 to 72 h post infection (hpi). Maximum attainable peak titers over a collection period of 96 h (addition of the amounts of viral particles in supernatant and cellular lysate, 1 ml each) in Vero cells for all SeV Fmut variants ranged between 7.6×10^6^ and 8.6×10^7^ TCID_50_, and in hepatoma cells between 6.2×10^6^ and 7.1×10^8^ TCID_50_ (Table 1).

**Table 1:** Peak titers in different cell types.

Cells	SeV Fmut	SeV FmutV_ko_	SeV FmutC_ko_	SeV FmutY_ko_	SeV FmutC_ko_V_ko_	SeV FmutC/Y_term_V_ko_
Vero	6.5×10^7^	8.6×10^7^	2.0×10^7^	2.5×10^7^	7.6×10^6^	9.2×10^6^
Hep3B	1.5×10^8^	1.3×10^8^	4.5×10^7^	1.7×10^7^	6.2×10^6^	1.4×10^7^
HuH7	1.1×10^8^	2.7×10^7^	2.5×10^7^	1.3×10^7^	9.3×10^6^	1.2×10^7^
PLC/PRF/5	7.1×10^8^	2.3×10^8^	5.4×10^7^	2.4×10^7^	3.2×10^7^	2.7×10^7^
MRC-5	7.9×10^5^	6.2×10^4^	2.0×10^4^	3.2×10^3^	8.0×10^3^	6.0×10^3^
PHH	2.8×10^6^	4.6×10^5^	4.0×10^4^	2.5×10^3^	3.8×10^3^	7.7×10^3^

Mean peak titers of resulting progeny virus of the six different SeV variants in Vero cells, three hepatoma cell lines (Hep3B, HuH7, PLC/PRF/5) and two non-malignant cells (MRC-5 and PHH). Numbers represent the mean of three independent experiments and are given as TCID_50_/2 ml.

In contrast to the malignant cell lines (Figure 3A), all SeV Fmut viruses showed a significantly reduced replication in two human-derived non-malignant cell types, the lung fibroblast cell line MRC-5 and in primary human hepatocytes (PHH) (Figure 3B). Over a collection period of 96 hpi maximum attainable peak titers now ranged between 2.5×10^3^ and 2.8×10^6^ TCID_50_ (Table 1). Interestingly, variants without C′/C or Y1/Y2 proteins produced less viral progeny than SeV Fmut or SeV FmutV_ko_.

Besides the effective production of viral progeny particles, we assume that the spreading within tumor tissues depends on both, the release of newly generated progeny virions, which theoretically can reach distant tumor cells, and the direct spreading to neighboring cells by a fusion of cellular membranes and syncytium formation. As shown in pictures of infected PLC/PRF/5 tumor cells (Figure 3C), nearly 100% of GFP expression (used here as a sign of viral infection) already displayed in the monolayer at 48 hpi for all SeV Fmut variants. In contrast, the F and P gene wild-type variant SeV D52 was not able to spread through the culture, which is due to its naturally restricted protease cleavage. At 72 hpi all SeV Fmut variant infections induced fluorescence negative areas within the cellular monolayer, being a result of massive tumor cell destruction. In contrast, the strongly reduced production of viral particles in non-malignant MRC-5 cells was in line with virus spread in these cells (Figure 3D, bottom panels). MRC-5 fibroblasts only allowed an effective spread throughout the culture for SeV Fmut or SeV FmutVko but SeV variants lacking C′/C or Y1/Y2 showed localized infection foci only (Figure 3D, panels to the right).

**Figure 4:**
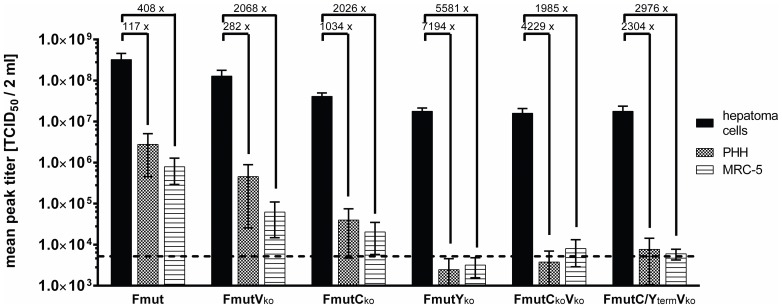
Comparison of attainable peak SeV titers during replication in different cell types. The mean peak titer (sum of the infectious virus particles determined via TCID_50_ for super natant and lysate) over a period of 96 h was determined including all experiments with the investigated three hepatoma cell lines (HuH7, Hep3B, PLC/PRF/5) and compared to the achievable peak titer in non-malignant cells (MRC-5 or PHH). Numbers over the columns display fold-changes between the mean of all hepatoma cells compared to either MRC-5 or PHH. The dotted line shows the amount of inoculated viral particles during the initial infection (5×10^3^ TCID_50_). Data represent mean and SEM.

In a direct comparison of peak titers (Figure 4) over a period of 96 hpi in human hepatoma cells (PLC/PRF/5, Hep3B and HuH7) versus human PHH or MRC-5 cells, a clear tumor selective replication was determined for the SeV Fmut knockout variants lacking the expression of full-length C/Y proteins with a low replication in non-malignant cells (up to 7,000-fold decrease). In particular, for SeV FmutY_ko_ the peak titers in non-malignant cells (MRC-5: 3.2×10^3^ TCID_50_; PHH: 2.5×10^3^ TCID_50_) did not even exceed the amount of particles used for primary infection (5×10^3^ TCID_50_) over a period of 96 h. We conclude, that no effective replication of SeV FmutY_ko_ took place in non-malignant cells, which was in clear contrast to the production of a high amount of viral progeny in hepatoma cells (mean peak titer for PLC/PRF/5, Hep3B and HuH7 hepatoma cells over the same collection period: 1.8×10^7^ TCID_50_).

**Figure 5:**
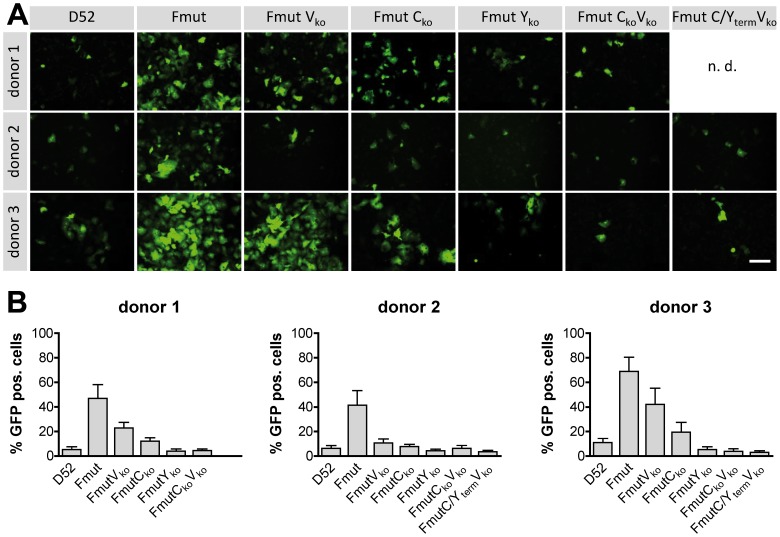
Attenuated SeV variants in primary human hepatocytes (PHH). PHH from three different donors were infected with SeV D52 and all six recombinant SeV variants (MOI of 0.1). **(A)** Exemplarily chosen pictures of PHH 72 hpi for seven different recombinant viruses (detection of GFP by fluorescence microscopy). Bar represents 200 µm. For donor 1, the analysis of the SeV FmutC/Y_term_V_ko_ variant was not done (n.d.). **(B)** 72 h after the inoculation with the different viruses, infected cells were counted in three randomly chosen areas and calculated as a ratio of all cells in the same area. Data are shown in mean and SD of three independent experiments in triplicates.

A strongly limited spreading of oncolytic viruses in tumor surrounding healthy cells is of utmost importance for the treatment of solid tumors. Therefore, we examined the GFP reporter gene expression and replication of all virus variants in detail in primary human hepatocytes as potential unwanted target cells for a virotherapeutic treatment of hepatocellular carcinoma (HCC) (Figure 5A). Infection experiments applying a MOI of 0.1 induced a GFP expression for SeV Fmut in 42 - 69% of PHH 72 hpi (determined ratio of counted GFP-positive cells; Figure 5B). Due to a lack of a F-protein specific protease in PHH, SeV D52 could only induce a primary infection but no spread, leading to 5–11% GFP positive cells. Notably, Fmut C_ko_, Fmut Y_ko_, Fmut C_ko_V_ko_, and Fmut C/Y_term_V_ko_ did not reach a much higher percentage of GFP positive cells than SeV D52. This supports our hypothesis that the strongly attenuated phenotype of variants lacking accessory proteins does not allow any efficient multi-round viral replication in non-malignant cell types.

### Cytolytic capacity of recombinant SeV particles in different cell types

**Figure 6:**
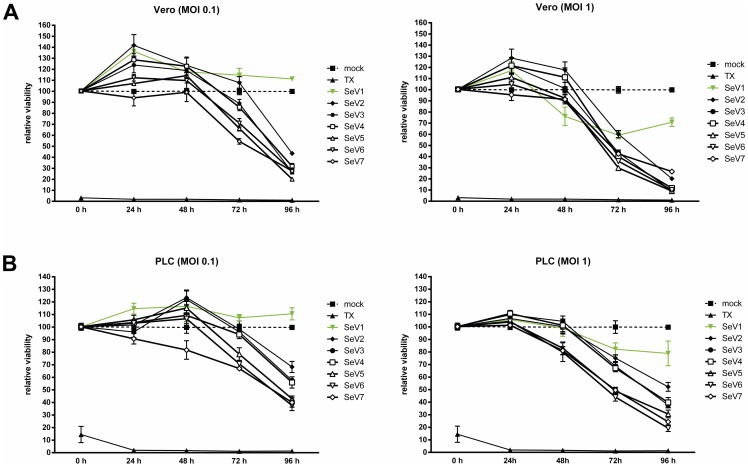
Quantification of cellular viability in a dose and time-dependent manner. Infection experiments with Vero and PLC/PRF/5 (at MOIs of 1 and 0.1) cells were performed with all variants of the newly generated Sendai viruses as well as the wild type variant SeV D52. Cellular viability was investigated via CytoTox-Glo™ assay at different time points post infection (0 h, 24 hpi, 48 hpi, 72 hpi, 96 hpi). The assay was performed in triplicates and repeated three times; data are shown as mean and SEM. SeV 1 (green line): SeV D52, SeV 2: SeV Fmut, SeV 3: SeV FmutV_ko_, SeV 4: SeV FmutC_ko_, 5: SeV FmutY_ko_, 6: SeV FmutC_ko_V_ko_, 7: SeV FmutC/Y_term_V_ko_, TX: Triton X-100 (positive control for the induction of a maximum grade, chemically-mediated destruction of test cells).

To investigate the influence on cellular viability in a time- and dose-dependent manner, Vero and PLC/PRF/5 hepatoma cells were infected by all variants of the newly generated Sendai viruses (at MOIs 1 and 0.1) as well as by the wild type variant SeV D52, used here as a P- and F-gene wild type control vector. At different time points post infection (0 h, 24 hpi, 48 hpi, 72 hpi, 96 hpi) cellular viability was tested using the CytoTox-Glow Assay™. As a result, a time- and dose-dependent loss of cellular viability could be shown for all new recombinant variants (Figure 6; black lines; SeV2-SeV7). In contrast, the wild-type variant SeV D52 which - due to its naturally restricted protease cleavage - is not able to spread through the cultures was found to exhibit only minor reductions in cellular viability (Figure 6; green line; SeV1).

**Figure 7:**
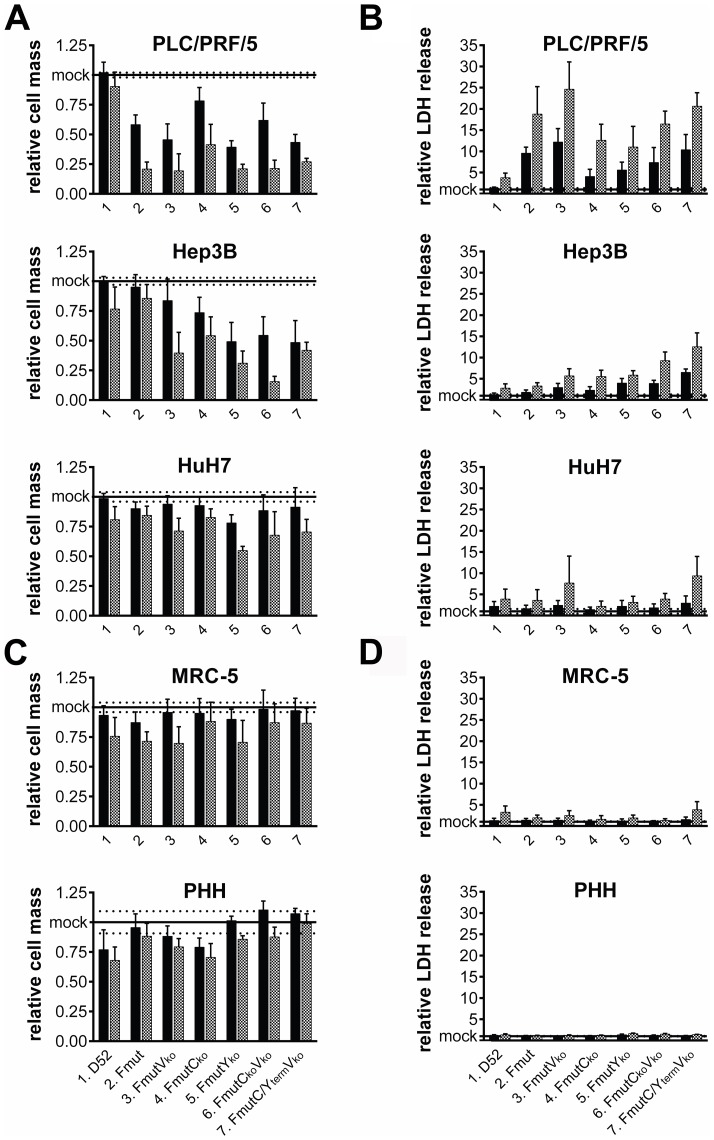
Cytolytic capacity of recombinant SeV particles in different cell types. Cell growth and cell lysis in different cell types with SeV D52 and six recombinant SeV Fmut variants (black bars: MOI 0.01, grey bars: MOI 0.1). (A+C) Analysis of cell mass by sulforhodamine B (SRB) assay 72 hpi in (A) hepatoma and (C) non-malignant MRC-5 cells or PHH. All values are shown in relation to uninfected control cells (mock). (B+D) Analysis of LDH release of infected cells relative to LDH release of uninfected control cells (mock) as a surrogate marker of cellular membrane integrity 72 hpi. Results represent mean and SD of three independent experiments in triplicates, for PHH two different donors, in triplicates each.

To further investigate the cytotoxic and especially the cytolytic potential in tumor cells we examined the cellular viability cell mass determined by a sulforhodamine B (SRB) assay as well as the loss of membrane integrity by determination of lactate dehydrogenase (LDH) release into the cellular supernatant 72 hpi. As a result, all hepatoma cell lines infected by the Fmut variants (MOI 0.01 and 0.1, respectively) showed a clear reduction of cell mass (Figure 7A). The most prominent results were observed for SeV variants FmutV_ko_, FmutY_ko_, FmutC_ko_V_ko_, and FmutC/Y_term_V_ko_. However, the different tumor cell lines showed a different level of response to infection with PLC/PRF/5 being the most sensitive cell line in this context. According to these results, the analysis of the percentage of LDH release into the supernatant (Figure 7B) was most prominent for PLC/PRF/5 cells for all of these viruses (11- to 25-fold change relative to baseline values of mock infections), whereas LDH release by HuH7 cells only displayed a slight elevation over baseline (between 2- and 9-fold; Figure 7B). Interestingly, the non-malignant fibroblast cell line MRC-5 and PHH from two different patient donors did neither show a comparable cell mass reduction, nor a relevant release of LDH into the supernatant (Figure 7C, D). Taken together, the attenuated rSeV Fmut variants clearly showed a tumor cell-specific destruction shown by the loss of cell mass or cellular lysis.

### *In vivo* safety of rSeV-knockout variants

As a prerequisite for a successful application of our newly generated rSeV particles *in vivo*, viral spread in tumor tissues was investigated in a murine model of human hepatoma xenograft with subcutaneously grown tumors of PLC/PRF/5 cells. Based on our *in vitro* studies, we chose to investigate the two most promising virus variants SeV FmutC_ko_V_ko_, or SeV FmutC/Y_term_V_ko_ and injected 1×10^7^ TCID_50_ of respective SeV variants directly into the tumors. As control viruses, we employed (i) SeV D52, which is not able to spread in this tumor tissue context due to the restrictive F protein cleavage-site, as well as (ii) SeV Fmut, which should facilitate an extensive spread without the presence of rare proteases. As wild-type SeV harbors a clear tropism for mouse cells, the xenograft model was very well suited to investigate both questions, efficient spread within the tumor and limited spread to distant tissues due to the engineered attenuation.

**Figure 8:**
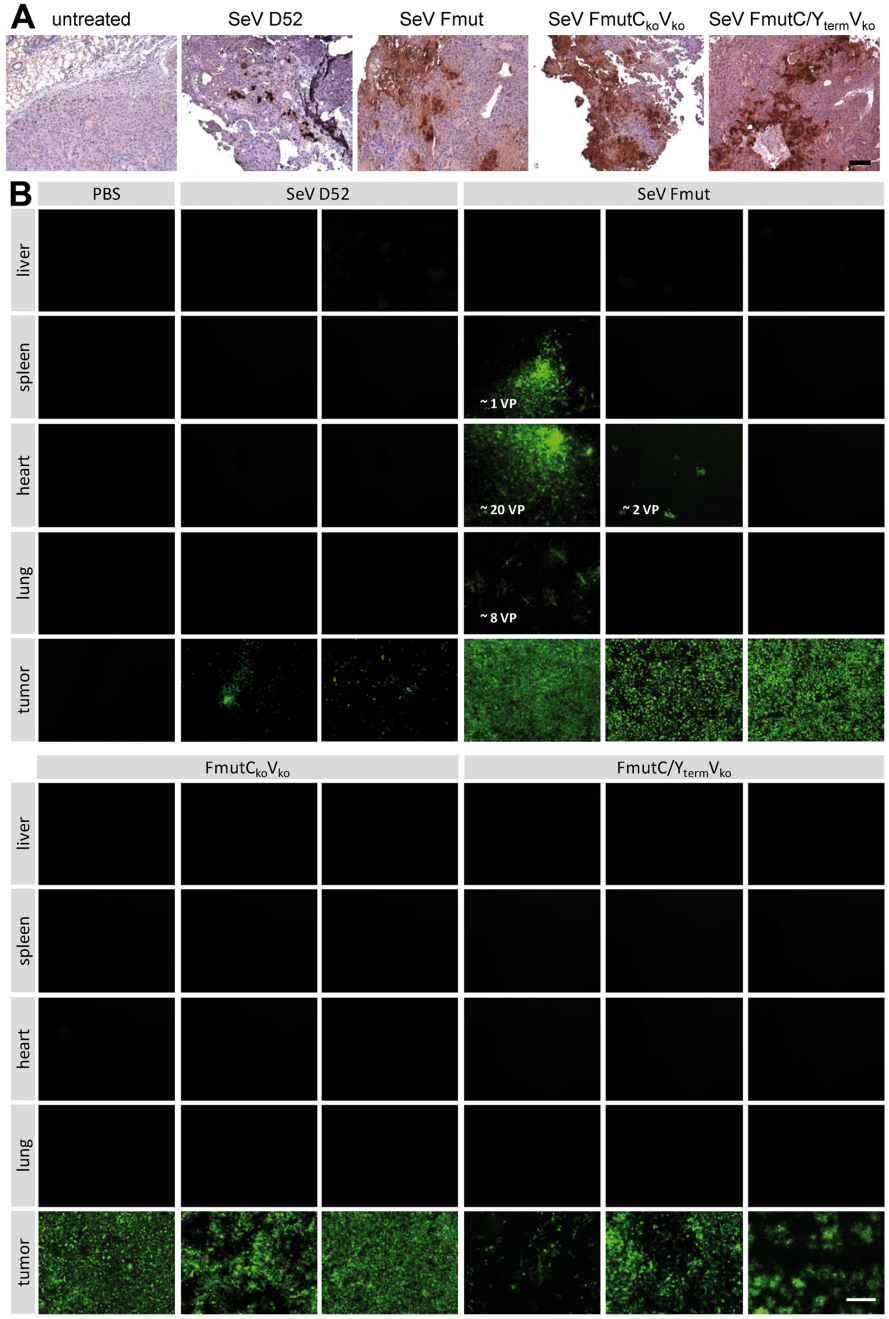
Intratumoral spread of recombinant SeV particles *in vivo* in a PLC/PRF/5 hepatoma xenograft model. Recombinant SeV variants (SeV D52, SeV Fmut, SeV FmutC_ko_V_ko_, SeV FmutC/Y_term_V_ko_, 1×10^7^ TCID_50_/100 µl) were injected intratumorally in tumors of a PLC/PRF/5 hepatoma xenograft mouse model. **(A)** 48 h post injection the mice were sacrificed, tumors were removed and one quarter of each tumor was fixed and embedded in paraffin. Virus spread was investigated applying an anti-GFP antibody for immunohistochemistry analysis (brown color). Bar represents 100 µm. **(B)** Indicator cultures (Vero cells) were infected with lysates from frozen tissue sections (one quarter of tumor, liver, spleen, heart, lung, each). Early primary infections (24-72 hpi) were observed and single infected cells were counted as initial virus particles (VP). Fluorescence microscopy pictures of the infected Vero cells were taken 72 hpi. Shown are representative picture for each animal. Bar represents 400 µm.

Two days after intratumoral virus injection tumor as well as liver, spleen, heart and lung were dissected and the tissue was analyzed for virus presence. In a histological staining of tumor samples for virus-encoded GFP, it became obvious, that SeV D52 induced only clearly distinguishable single infection foci (Figure 8A, second panel from the left). In contrast, all viruses with an altered F protein cleavage-site (Fmut) were able to spread throughout the tissue over a distance of more than 100 µm (Figure 8A, panels to the right).

**Figure 9:**
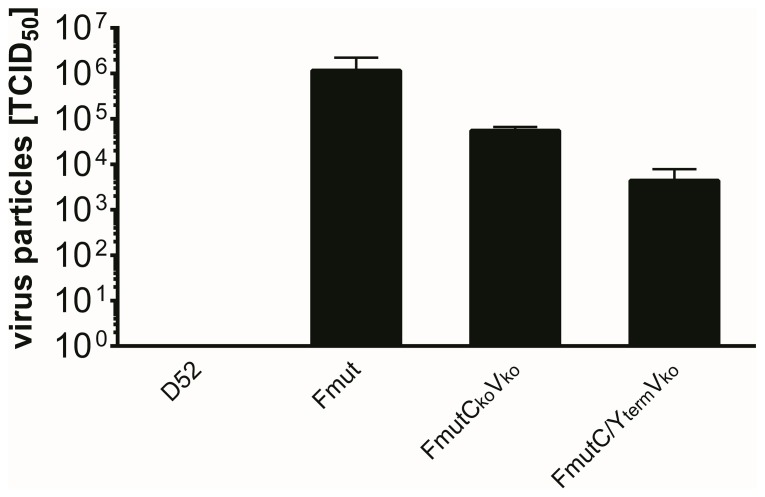
The amount of infectious virus particles in tumor tissue was quantified with the TCID_50_ method. 48_50_ titration (mean and standard deviation of two (D52) or three analyzed tumors of each group).

Next, samples (1/4 each) from the xenograft tumors as well as from liver, spleen, heart and lung were analyzed (i) for the pattern of intratumoral spreading of our rSeV variants (Figure 8A), (ii) for the presence / absence of our rSeV variants in tumorous versus non-tumorous tissues (i.e., tumor, liver, spleen, heart, and lung) (Figure 8B), and (iii) for the amount of infectious virus particles being present in the fine needle-addressed tumor tissues at 48 hours after virus injection (Figure 9).

As expected, infectious particles of all three virus rSeV variants containing the Fmut protease cleavage-site could be isolated at high levels from the tumor tissues (Figure 8B, Figure 9), whereas SeV D52 infectious particles could only be isolated from tumor tissue at a quite low level (Figure 8B, upper panel; Figure 9, left bar). For the SeV Fmut variant expressing all accessory proteins, the expanded tissue tropism could be documented by the detection of single infectious particles in spleen, heart and lung tissues (Figure 8B, upper panel). In contrast to SeV Fmut, for the attenuated viruses (Figure 8B, lower panel) no infectious particles were detected in non-tumorous, normal tissues (employed by a very sensitive virus amplification assay based on Vero indicator cell cultures), a finding which clearly documents the high level of selectivity of these viruses for tumor cells in vivo (Figure 8B).

## Discussion

SeV particles as potential antitumor agents harbor an attractive profile including a broad host range due to ubiquitously available sialic acid residues that serve as cellular receptors and ensure a rapid uptake into the cytoplasm of host cells within minutes. Additionally, infections are cytotoxic, which is at least in part mediated by a pronounced syncytium generating activity [11], [41].

The aim of this study was to develop a new generation of Sendai viruses exhibiting profound oncolytic activities while showing a highly attenuated phenotype in non-malignant cells. Efforts have already been undertaken to engineer SeV for a tumor application, but in contrast to our strategy, the so far reported anticancer effect was limited twofold: (i) it was dependent on the secretion of tumor cell-specific proteases such as urokinase-type plasminogen activator [24], [25] or matrix metalloproteinases [23], and (ii) spread of viruses did only occur by fusion events with neighboring cells due to a lack of progeny release via the cellular membrane. Thus, all these approaches were strictly limited to the injection site and to a preselected small subset of tumor types.

As a proof-of-concept study we here show that a new generation of SeV particles with a different safety concept rapidly and selectively spreads in tumor cells *in vitro* as well as in tumor tissue *in vivo*. This new phenotype could be achieved by a two-step approach: First, a broadening of the tissue restriction by introduction of a cleavage-site into the F protein that is recognized by ubiquitous furin-like proteases. Second, a limitation of viral replication in non-malignant cell types was achieved by preventing the expression of accessory regulatory proteins (C′, C, Y1, Y2, V, W). These accessory regulatory proteins of SeV are e. g. known to interfere with molecules of the IFN type I system and thus suppress the establishment of an antiviral state in virus infected cells [36]. In generating rSeV Fmut C/Y/V knockout variants we expected a reduced spread and replication in IFN competent non-malignant cells, similar to IFN-sensitive VSV viruses [7] or oncolytic measles viruses with defects in IFN-antagonizing proteins [5]. As a matter of fact, we observed a highly reduced replication especially for rSeV knockout variants without C and Y proteins in non-malignant MRC-5 cells and PHH while keeping the ability to replicate to high titers and spread in hepatoma cells. These observations may be due to the fact, that hepatoma cells are known to have defects at several steps in the IFN signal transduction [42], [43], [44], whereas non-malignant cells are able to induce an antiviral state limiting the virus replication and thus virus-induced cytotoxicity. Beyond that, also other factors, such as reduced rates of (i) primary target cell infection and / or (ii) target cell progeny virus particle release, could have been contributed to the observed attenuated phenotype of our rSeV Fmut C/Y/V knockout variants. Especially in the context of any future clinical application it is expected that kinetic differences of the distinct phases of viral replication of our rSeV variants in malignant versus non-malignant cells (i.e., primary target cell infection, target cell replication, and target cell progeny virus particle release) will contribute to differences in vector spreading and replication. However, up to now there are no means to discriminate major from minor factors of attenuation in a clinical context.

Together with a recent report that SeV vectors have for the first time reached the clinics for therapeutic gene transfer [17], our backbone modifications open up the perspective to investigate SeV particles also in clinical oncology. The destruction of tumor cells by virus-induced apoptosis [45], [46] or syncytia formation by SeV add new target mechanisms to the already existing therapeutic tools that are currently applied in routine patient treatment. Thus, virotherapeutics may be applied in the future to overcome resistance phenomena to conventional therapeutic strategies or even may be ideal combination partners to already existing therapeutic approaches [2], [3], [5]. Of note, a direct and detailed comparison of different oncolytic vector types in a given tumor entity has not been performed yet, but it can be expected that for each tumor type or even subtype an optimal virotherapeutic approach has to be developed separately, defining the most promising virotherapeutic strategy for clinical routine application.

As hypothesized, our data clearly show a tumor selective replication of the newly generated SeV Fmut variants in different cell types *in vitro*. As SeV has a broad host range, which opens up the possibility to apply SeV-based virotherapeutics in the context of different cancer types, the focus of our study did not intend to show efficacy in a selected specific tumor model. In fact, our aim was to demonstrate that SeV can be employed as a tool to selectively target tumor tissues, followed by a distribution therein. In our exemplarily selected HCC xenograft model we found a spread of SeV within tumor tissue, which was dependent on the introduction of the modified SeV cleavage-site (Fmut). Thus, the protease-restriction of SeV wild-type, which strictly limits the virus spreading to the respiratory tract, where SeV specific proteases are present, could be overcome. Concerning biodistribution of our new vector type, in a highly sensitive assay for detection of infectious particles we found a tissue restriction to the tumor site for all attenuated virus variants. Infectious particles could not be detected within samples from organs of mice after intratumoral application of SeV, including liver, spleen, heart and lung. Especially in the case of a direct (i.e., fine needle-bound) intratumoral virus application in the preclinical setting, contact of virotherapeutics only is made to tumor areas directly surrounding the needle tract, resulting in a strictly tumor-bound colonization with virotherapeutics. Similarly, in the clinical context only minor contacts, to non-tumorous areas (localized immediately adjacent to virotherapeutically addressed tumors) are established in the course of virus injection. Therefore, presence of in-patient foci exhibiting high MOIs in normal tissues (amount of virus particles locally outnumbering the amount of neighbouring normal cells) is supposed to constitute an exemption. This setting of “low normal tissue MOIs” has to be considered even more true for the intravenous and intracavital (intraperitoneal, intrapleural) routes of virus application in which a quite rapid and profound dilution of virus particles takes place through the blood stream or through contact with intraperitoneal or intrapleural fluids.

The value of a selectively replicating virus within tumor tissue could be recently shown in a clinical pilot study in which the systemic injection of an oncolytic poxvirus in patients induced a profound infection of tumor tissues with the expression of virus encoded transgenes [47]. This implies that tumor selective viruses are not only able to use tumor cells as host cells; moreover, they might even be applied to express high concentrations of therapeutic or imaging proteins in solid tumors in humans [48], [49]. Therefore, our newly generated tool of oncolytic SeV particles will have to be tested in different tumor models in the near future. Additionally, it will be possible to generate enhanced “armed” rSeV variants which express prodrug converting enzymes or immunostimulatory molecules, such as cytosine deaminase [50], [51] or GM-CSF [52] within their viral backbones.
